# Dr. Alice M. Potter

**Published:** 1903-07

**Authors:** 


Dr. Alice M. Potter, University of Buffalo, 1897, of Ithaca, died May 2, 1903, of typhoid fever, aged 33 years. It appears that Dr. Potter fell a victim to the epidemic that she sought to stay by her self-sacrificing devotion. Her case was complicated by appendicitis, for which an operation was made. Appropriate action was taken upon her death at a special meeting of the physicians of Ithaca.

